# Récidive après dix ans de tumeur de granulosa de l’ovaire: à propos de deux cas et revue de la littérature

**DOI:** 10.11604/pamj.2016.25.30.10433

**Published:** 2016-09-27

**Authors:** Soufya Majdoul, Nezha Tawfiq, Zouhour Bourhaleb, Nora Naqos, Amina Taleb, Zineb Bouchbika, Nadia Benchakroun, Hassan Jouhadi, Souha Sahraoui, Abdelatif Benider

**Affiliations:** 1 Service de Radiothérapie, Faculté de Médecine et Pharmacie, Université Hassan II de Casablanca, Maroc; 2 Service d’Oncologie Médicale, Faculté de Médecine et Pharmacie, Université Hassan II de Casablanca, Maroc; 3 Centre Mohammed VI de Traitement des Cancers, Faculté de Médecine et Pharmacie, Université Hassan II de Casablanca, Maroc

**Keywords:** Tumeur de granulosa de l´ovaire, récidive tardive, traitement, Granulosa cell tumors of the ovary, late recedivism, treatment

## Abstract

Les tumeurs à cellules de la granulosa (TG) de l’ovaire sont des tumeurs rares appartenant au groupe des tumeurs des cordons sexuels et du stroma. Elles représentent 0,6 à 3 % de l’ensemble des tumeurs de l’ovaire et 5 % des tumeurs malignes On distingue deux types: le type juvénile (TGJ) qui se caractérise par son agressivité et le type adulte (TGA) qui est le type le moins agressif et le plus fréquent. Les rechutes de TG de l'ovaire surviennent généralement dans les cinq ans, elles sont rarement sous forme de métastases péritonéales ou locales. Bien que, des options de traitement y compris la chirurgie avec ou sans chimiothérapie et ou radiothérapie ont été rapportés pour le traitement des récidives des TG, il n'y a aucune prise en charge standardisée de récidive de cette maladie. Ici, nous rapportons notre stratégie thérapeutique dans la prise en charge des récidives tardives, après dix ans, de la TG sous forme de carcinose péritonéale pour deux patientes avec une revue de la littérature.

## Abstract (en)

Granulosa Cell Tumors (GCT) of the ovary are rare tumors belonging to the group of sex cord stromal tumors. They represent 0.6 - 3% of all ovarian tumors and 5% of all malignant tumors. Two different types of GCT can be distinguished: juvenile granulosa cell tumor (JGT) which is characterized by its aggressive potential and adult granulosa cell tumor (AGT) which is the most common and the least aggressive type. GCT of the ovary usually develops recurrences within 5 years of initial diagnosis but they rarely develop local or peritoneal metastases. Although treatment options including surgery with or without chemotherapy or radiation have been reported in treating GCT relapses, there are no standardized protocols for the treatment of relapses. We here present our therapeutic strategy in the treatment of longterm relapses of GCT peritoneal carcinomatosis occurring in two patients 10 years after the initial diagnosis and a review of the literature.

## Introduction

Les tumeurs de la Granulosa représentent 5 % des tumeurs malignes de l’ovaire et sont les plus fréquentes des tumeurs des cordons sexuels et du stroma avec une incidence de 0,58 – 1,6/ 100 000 femmes par an [1, 2]. La forme adulte (TGA) est la plus fréquente (95 %) [1, 3]. Stimulées par l’hormone folliculo-stimulante (FSH), les cellules de la Granulosa permettent la croissance folliculaire et sont responsables d’une hyperestrogénie par la production d’estrogènes, l’hormone anti-Müllerienne (AMH) et l’inhibine B. Elles surviennent en période péri et post-ménopausique avec un pic de fréquence autour de 50 à 55 ans [3–5]. La forme juvénile est beaucoup plus rare représentant 5 % de ces tumeurs. Elle survient chez des femmes jeunes moins de 30 ans ou en période pré-pubertaire [3, 4]. L’évolution des TGA est lente et les récidives sont souvent rares et tardives, survenant dans les 5 à 6 ans [2, 3]. Nous rapportons deux cas de métastases péritonéales tardives après dix ans de fin de traitement d’une tumeur à cellules de Granulosa de l’adulte.

## Patient et observation

### Observation 1

Patiente âgée de 41 ans, sans antécédents pathologiques particuliers, a consulté en 1999 pour des douleurs abdominales aigues. La TDM thoraco-abdomino-pelvienne avait objectivé une masse kystique multi-cloisonée de 8 cm de l’ovaire gauche. L’intervention a consisté en une kystectomie gauche. L’examen anatomopathologique a trouvé une tumeur de Granulosa. La patiente a été reprise pour annexectomie gauche. L’analyse anatomo-pathologique n’a pas montré de malignité. Elle a reçu 6 cures de chimiothérapie adjuvante.

**Figure 1:**
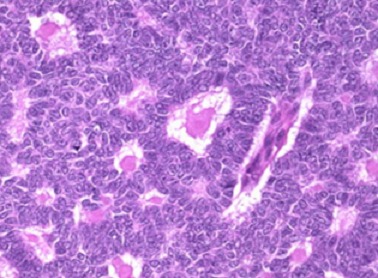
Métastases péritonéales de tumeur de la granulosa sous forme de masses solido kystiques

Dix ans après, la patiente a consulté pour des douleurs de la fosse iliaque droite. La TDM abdomino-pelvienne a objectivé une masse de l’ovaire droit de 8 cm kystique. Elle a bénéficié d’une HTSCA avec omentectomie. L’examen anatomo-pathologique a révélé une tumeur de la granulosa de l’ovaire droit avec envahissement de la paroi tubaire droite et localisation péritonéale.à l’immunomarquageétait négatif au CK7 et positif de façon massive à l’inhibine (Figure 1). La TDM thoraco-abdomino-pelvienne post-opératoire n’avait pas montré de résidu tumoral macroscopique. Le CA 125 était normal. Elle a reçu 6 cures de chimiothérapie adjuvante associant le cyclophosphamide et le cisplatine.

**Figure 2:**
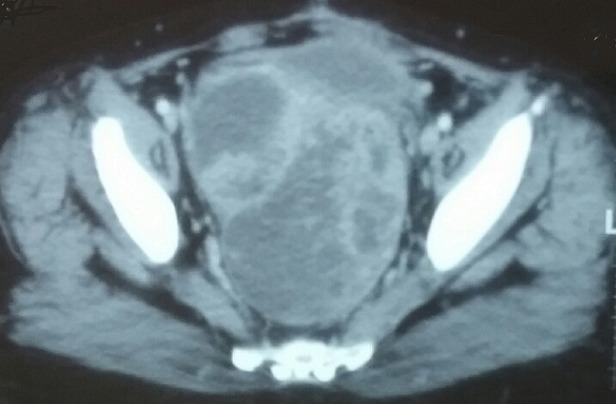
Coupe axiale: montrant la récidive péritonéale de tumeur de Granulosa

En 2011, suite à des douleurs pelviennes, une masse solido-kystique rétro-vésicale de 12x9.8x5 cm sans liseré de séparation avec la vessie a été diagnostiquée sur le scanner abdominopelvien (Figure 2). Lors de la laparotomie, la masse retro-vésicale était inextirpable. L’examen anatomopathologique a confirmé la récidive péritonéale de la tumeur de granulosa type adulte. Une chimiothérapie de 2éme ligne a été indiquée à base de Taxol/ carboplatine en 6 cycles. L’évaluation a montré une stabilisation des lésions radiologiquement et une amélioration clinique de la patiente. Une pause thérapeutique avec surveillance rapprochée a été adoptée. La patiente était régulièrement suivie en consultation, jusqu’au 2013, suite à des douleurs abdominales, un scanner abdominal a objectivé une augmentation de la taille de la masse de cul de sac de douglas. La patiente a été mise sous Anti Aromatase pendant deux ans. La tolérance clinique était excellente. L’évaluation montrait une amélioration des symptômes et une diminution régulière de la taille et le nombre de l’ensemble des nodules péritonéaux.

En février 2015, l’imagerie a montré une augmentation de la taille de la masse du cul de sac de douglas avec l’apparition d’une masse inter-hépato diaphragmatique, la carcinose péritonéale. Elle a été mise sous Bevacizumab à la dose de 7,5mg/m2. L’évaluation a montré une stabilité des nodules péritonéaux selon les critères RECIST.

### Observation 2

IL s’agit d’une patiente âgée de 41 ans, multipares 3 gestes 3 pares, ayant un antécédent de laparotomie en 1992 pour masse ovarienne gauche dont l’examen anatomopathologique a montré une tumeur de granulosa de l’ovaire. Douze ans plus tard, la patiente a eu une 2éme intervention : HTSCA pour tumeur ovarienne droite l’examen anatomopathologique a conclut une tumeur de Granulosa, une chimiothérapie adjuvante était administré à base de cyclophosphamide et cisplatine.

En 2004, elle s’est présentée à la consultation pour une douleur abdominale associée à une masse de FID. La TDM a montré une masse para-ceocale de 10 cm. Les marqueurs tumoraux (CA 125, CA19-9 et ACE) étaient normaux. Elle a eu une exérèse de cette masse et une omentectomie. A l’examen anatomo-pathologique, il s’agissait d’un kyste séro-hématique rétentionnel à paroi dissociée par des reliquats néoplasiques associé à une localisation épiploïque d’un carcinome peu différencié dont le l’immunohistochimie a conclu une tumeur de granulosa de l’ovaire. Elle a été régulièrement suivie en consultation par échographie abdomino-pelvienne et dosage du CA 125.

**Figure 3:**
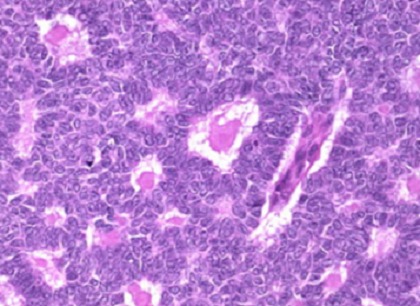
Récidive de tumeur de granulosa de l’ovaire sous forme d’une masse rétro vésicale solido kystique multi cloisonnée de 12x9x5cm

En 2010, l’échographie a objectivé une masse kystique multi-cloisonnée de 59 x 47 mm en retro-vésical. La TDM abdomino-pelvienne a montré la masse rétro-vésicale gauche de 54 x 44 mm rehaussée de façon hétérogène avec le produit de contraste et contenant une nécrose centrale. Le CA 125 était normal. En per-opératoire la masse n’était pas résècable de façon carcinologique. Elle a eu de simples biopsies. L’étude anatomo-pathologiquea permis de retenir le diagnostic de tumeur de la Granulosa de l’ovaire. Une chimiothérapie à base de paclitaxel et carboplatine a été administrée. L’évaluation a montré une rémission complète radiologique. En 2014, la patiente a présenté sur l’échographie abdomino-pelvienne deux nodules hépatiques et des nodules péritonéaux. Le scanner a confirmé la présence de multiples masses péritonéales en péri-hépatique et des nodules péritonéaux (Figure 3). A l’étude histologique, il s’agissait d’une localisation épiploique et péritonéale d’une prolifération glandulaire peu différenciée invasive compatible avec une origine ovarienne. La patiente a été mise sous anti-aromatase. Après 9 mois de traitement, la patiente était asymptomatique avec un aspect stable des lésions au scanner.

## Discussion

Les TG ont été décrites pour la première fois en 1855 par Rokitansky par leur aspect proche de celui des cellules de la granulosa du follicule ovarien [1]. Elles appartiennent au groupe des tumeurs du mésenchyme et des cordons sexuels, et représentent plus de 70 % des tumeurs malignes de ce groupe et 5 % des cancers de l’ovaire [6, 7]. L’incidence de cette tumeur était estimée par Lauszuset al. [6] à 1,3 par an et par100 000 femmes. On distingue deux formes anatomo-cliniques: la forme adulte (95%) qui survient le plus souvent entre 40 et 70 ans et la forme juvénile (5%) qui survient souvent avant l’âge de 20 ans [8]. Elles ont un faible degré de malignité et sont de bon pronostic avec toutefois une tendance plus agressive dans la forme juvénile [3, 4]. Les récidives tardives après 10 ans sont rares [9]. Les récidives de tumeur de Granulosa surviennent en général dans les cinq ans. Deux cas ont été rapportés dans la littérature [9, 10]. Nous rapportons deux cas de récidives tardives après dix ans.

Cliniquement la tumeur se manifeste par un syndrome tumoral avec distension abdominale douloureuse, un syndrome endocrinien lié à une hyper-oestrogénie donnant une pseudo-puberté précoce iso sexuelle dans les formes juvéniles. En période péri ménopausique, cette hyper-oestrogénie explique l’hyperplasie endométriale qui peut être atypique.

Le diagnostic préopératoire en imagerie des tumeurs des cellules de la granulosa est difficile compte tenu de leur grande variabilité morphologique. L’échographie et le scanner pelvien ont montré pour nos patientes un aspect en faveur d’une tumeur mixte solido-kystique non spécifique. Selon Mancaux et al, l’approche diagnostique radiologique des tumeurs de la granulosa repose sur l’échographie pelvienne couplée à l’IRM. Elles se présentent, le plus souvent sous forme d’une masse latéro-utérine de grande taille, solido-liquidienne, unilatérale, avec composante hémorragique intra tumorale sans calcification, graisse ou végétation, parfois avec une ascite ou des nodules péritonéaux. Néanmoins, étant donné leur grand polymorphisme, l’imagerie médicale ne permet pas de donner une définition précise. Devant une imagerie évocatrice de tumeur de la granulosa, il convient de réaliser des explorations hormonales comprenant un dosage de l’inhibine B (le marqueur le plus fiable des TG), l’hormone anti-Müllerienne (AMH), l’estradiol et la protéine de régularisation folliculaire (FRP). Le géne Foxl2, codant un facteur de transcription impliqué dans l’embryogenèse, la cancérogenèse et la différenciation cellulaire, est porteur dans 97 % des TGA d’une mutation faux-sens (C402G; Cys134Trp), qui semble être une piste diagnostique prometteuse et un probable futur marqueur d’efficacité thérapeutique et de récidive.

La chirurgie est le traitement de référence. Elle permet une stadification de la maladie et consiste classiquement en une hystérectomie totale, annexectomie bilatérale, et une omentectomie. Le curage ganglionnaire ne semble pas améliorer la survie pour les stades précoces [1]. Selon Shim et al, la voie cœlioscopie semble sure et faisable pour ce type de tumeur [9]. Un traitement conservateur est parfois proposé aux femmes jeunes désireuses de grossesse avec un stade I comprenant l’annexectomie unilatérale, l’exploration de la cavité abdomino-pelvienne et la biopsie endométriale.

Le diagnostic définitif des tumeurs à cellules de la granulosa est anatomopathologique. Macroscopiquement, ces tumeurs sont décrites comme une tumeur solide, multi cavitaire. Au plan histologique, ce sont des tumeurs de l’ovaire constituées des cellules de la granulosa, d’une composante de fibroblastes et de cellules thécales responsables des manifestations endocrines oestrogéniques et plus rarement androgéniques permettant un diagnostic clinique précoce et une prise en charge rapide [7]. Les corps de Call Exner, typiques dans la tumeur sont des petites zones arrondies de liquide extracellulaire et de débris cellulaires, entourées de cellules de la granulosa bien différenciées, organisées en rosettes autour de ces petites zones. Les cellules ont un aspect en grain de café [8].

Des traitements adjuvants peuvent être proposés en cas de stade III et IV ou en cas de récidive : chimiothérapie type PVB, BEP ou carboplatine + paclitaxel [1], radiothérapie pelvienne et/ou abdominale [2] voire hormonothérapie par anti-aromatase AI [7, 10]. Différents protocoles ont été étudiés ces 40 dernières années. Tout d’abord les monochimiothérapies à base de cisplatine et de cyclophosphamide, Ces derniers ont montré une activité modeste. Puis des associations thérapeutiques à base de doxorubicine et de cisplatine ont permis d’obtenir 17 réponses objectives sur 27 cas traités pour TGO dans les séries publiées [11]. L’activité et la tolérance du BEP ont été confirmées dans une étude phase II du groupe GynecologicOncology Group (GOG) [11]. Récemment, le BEP a été comparé aux taxanes dans une étude chez des patientes avec TGO. L’activité et la tolérance ont été similaires ref. Cependant, les taxanes sont plus réservés en deuxième ligne en monothérapie ou en association aux sels de platine [12].

Selon Haupsy et al. [11], la radiothérapie postopératoire augmenterait la survie sans récidive (251 mois versus 112 mois, p = 0,02). Pour Park et al. [1], la chimiothérapie (BEP) serait un facteur protecteur pour les stades avancés (p = 0,022). Il n´y a aucune prise en charge standardisée de récidive. Une étude publiée par Canbay et al a montré un bénéfice de la chirurgie de réduction associée à une chimiothérapie intra-péritonéale hyperthermique peropératoire utilisant le cisplatine 100 mg pendant 40 min à 43 ° C en cas de métastases péritonéales [13]. Le rôle de la radiothérapie en cas de maladie résiduelle post opératoire est controversé, mais les différentes études rétrospectives rapportées dans la littérature ont montré un taux de réponse complète de 43- 50% [14, 15].

Pour la thérapie ciblée, Schmidt et al. ont trouvé que 94 % des patientes avec TGO expriment le VEGF. De même que Brown et al. ont montré que l’augmentation de la densité microvasculaire et la surexpression du VEGF sont corrélées à la présence de métastases à distance. L’analyse rétrospective concernant huit patientes (7 : TG adulte ; 1 : TG juvénile) traitées par Bevacizumab pour une récidive a noté une réponse complète, deux réponses partielles, deux stabilisations et trois progression un taux de réponse de 38 %, un taux de bénéfice clinique de 63 % et une médiane de survie sans progression (SSP) à 7,2 mois [12]. Une de nos patientes a eu le bévacizumab avec une excellente tolérance et une réponse clinique après 6 cycles.

L’hormonothérapie à base des antiromatases AI a montré un bénéfice selon les trois études rétrospectives publiées [12]. Seules sept patientes avec TGO récidivantes traitées par des AI sont rapportées dans la littérature. Toutes les patientes ont eu une réponse clinique dépassant 12 mois, dont une patiente avait une DFS de 54 mois. Nos deux patientes ont bénéficié d’un traitement par AI avec une réponse clinique de 12 et 24 mois.

Selon la littérature, les facteurs de risque de récidive sont : stade FIGO avancé, la présence de tumeur résiduelle, la taille tumorale > 13,5 cm [16]. Pour les stades I–II, la survie sans récidive est de 89 % et la survie globale de 90 % à 10 ans. Pour les stades III–IV, elle est respectivement de 67 % et 57% [1]. Selon Li et al. [10], le stade FIGO, les atypies nucléaires et l’index mitotique seraient des facteurs de risque de décès et la rupture tumorale un facteur de risque de récidive.

La chimiothérapie adjuvante et la réalisation d’un staging chirurgical complet augmenteraient la survie sans récidive selon Mangili pour les TG de haut risque de récidive.

## Conclusion

La tumeur à cellules de la granulosa est une tumeur rare et lentement progressive. Elle s’inscrit dans le cadre des diagnostics différentiels de l’adénocarcinome ou des carcinomes indifférenciés de l’ovaire. Elle a un pronostic nettement moins réservé, d’autant qu’il s’agit le plus souvent d’un stade I pour lequel la survie à 10 ans est de 84 à 95 %. Le traitement repose sur la chirurgie, d’emblée radicale pour les femmes âgées, associant une hystérectomie totale avec une annexectomie bilatérale, une omentectomie et des biopsies péritonéales. Toutefois, une annexectomie avec curetage biopsique de l’endomètre est proposée pour des femmes jeunes de stade Ia, désireuses de grossesse. La chimiothérapie (BEP ou BVP) est indiquée dans les formes avancées, les récurrences ou les métastases. L’intérêt de la radiothérapie est discuté. Des essais thérapeutiques par hormonothérapie ont été rapportés. Les anti-aromatases ont permis d’avoir une stabilisation assez prolongée et de retarder le recours à la chimiothérapie. Leur utilisation trouve son rationnel dans l’hormonosensibilité démontrée par les études rétrospectives, la durée de réponse et la meilleure tolérence comparée à la chimiothérapie. Le bevacizumab a démontré son efficacité dans les tumeurs épithéliales de l’ovaire. Son utilisation dans les formes avancées et les rechutes des tumeurs de la granulosa devrait être utilisée à plus grande échelle d’autant plus qu’elles sont riches en VEGF. Du fait de la faible incidence des TGO, des essais prospectifs ne peuvent être réalisés. Vu les récidives tardives, nous encourageons la surveillance prolongée et l’utilisation des thérapies ciblées tel les anti-aromatases et les anti-VEGF dans les rechutes pour avoir plus d’arguments pour les introduire dans le traitement adjuvant.
